# Advances in Molecular Research of Oncogenes

**DOI:** 10.3390/ijms24087222

**Published:** 2023-04-13

**Authors:** Fernando C. Baltanas, Eugenio Santos

**Affiliations:** 1 Centro de Investigación del Cáncer, Instituto de Biología Molecular y Celular del Cáncer, CSIC-University of Salamanca and CIBERONC, 37007 Salamanca, Spain; 2 Departamento de Fisiología Medica y Biofísica, Universidad de Sevilla, 41001 Sevilla, Spain

The isolation of the first human oncogene (HRAS), a critical breakthrough in cancer research, has occurred over forty years ago, and the identification of new pathogenic oncogenes has continuously grown since. The detailed mechanistic characterization of the way in which oncogenes dysregulate physiological signaling to trigger different cancer types and developmental syndromes, as well as the discovery of specific small-molecule inhibitors targeting the different oncogenic proteins, are potential aspects to forward our understanding of cancer research in the following years. The purpose of this Special Issue was to explore the expanding field of molecular research of oncogenes, so as to provide valuable translational indicators that could potentially meet clinical needs.

This Special Issue comprises four research articles and six reviews. The research articles describe new and interesting methodological approaches, identify feasible therapeutic targets for certain tumor types, and unveil a new small molecule able to inhibit a specific oncogene. In particular, Abuasaker and colleagues [1] described a novel small molecule (P14B) that stably binds to a druggable site of the allosteric domain of KRAS, the most frequently mutated oncogene in a wide spectrum of tumor types. Importantly, the viability of tumor cells, but not that of normal cells, was the only impaired factor upon P14B administration, supporting the significance of the α4–α5 helical regions of KRAS in the regulation of oncogenic KRAS-dependent diseases, and additionally demonstrating tolerance to its administration. 

Chen et al. [2] proposed the phosphatidylethanolamine binding protein 4 (PEBP4), an understudied multifunctional protein, as a potential therapeutic target for hepatocellular carcinoma. Consistent with this notion, the authors observed that the increased expression of PEBP4 directly correlated with the increased malignancy of the hepatocellular cancer cells. In addition, they unveiled mechanistic insights of this behavior indicating a cooperative participation of mTORC1 and mTORC2-mediated signal transmission in the process. 

With regards to the methodological articles, Kopra et al. [3] described a new methodology to evaluate and characterize newly designed drug candidates. The authors designed a protocol improving previously established methodologies, of which the sensitivity enables effective studies with non-covalent and covalent drugs at the nanomolar level. 

Vuelta and co-workers [4] presented a gene-trapping approach combined with CRISPR technology as an ideal methodology to increase the selection of CRISPR-edited cells as a potential approach for the treatment of BCR/ABL-driven chronic myelogenous leukemia (CML). Strikingly, both in vitro and in vivo experiments demonstrated a significant reduction in CML tumor cell proliferation and tumor growth, thus providing a proof-of-concept for the therapeutic potential of a CRISPR-trap system as a novel strategy for gene elimination in hematological neoplasms. 

A total of six reviews revisiting different aspects of cancer research (new therapeutic targets or diagnostic markers, improvement of methodologies or deficiencies in the accessibility of precision oncology worldwide) were included in this Special Issue. In this regard, Alcoceba et al. [5] summarized the clinical applications of *MYD88* and its specific mutation, *MYD88^L265P^*, in the diagnosis, prognosis, follow-up, and treatment of patients with lymphoma. Sobolev and co-workers [6] reviewed the physiological relevance of the FOSL1 gene and its role in solid tumors, the importance of FOSL1 as a prognostic factor, and the perspectives of this gene as a molecular target for anticancer therapy. Kasprzak [7] revised the role of the ghrelin system in colon carcinogenesis, diagnostics, and colorectal cancer prognostics. Chenais made an intricate summary of the current knowledge about transposable elements and how they can be employed as new potential diagnostic markers of diseases, particularly cancer [8].

Miron-Mombiela et al. [9] pointed out the importance of imaging as an emerging field in cancer research, establishing an association between radiological features and genomic/molecular expression patterns to explain underlying disease mechanisms and enhance prognostic, risk assessment, and treatment response radiomics models in cancer patients.

Finally, Marima et al. [10] focused on the important, yet frequently underrated, aspect of global disparities in medicine, especially precision oncology, due to the lack of adequate genetic information or databases from the African genome. This review focuses on the high mortality rates associated with type II endometrial cancer in black women and prostate cancer in men of African ancestry, and the importance of properly establishing an entire transcriptomics landscape in an African cohort that may help elucidate the relationship between race and pathological disparities of these two diseases.

## References

[1] Abuasaker B., Garrido E., Vilaplana M., Gómez-Zepeda J.D., Brun S., Garcia-Cajide M., Mauvezin C., Jaumot M., Pujol M.D., Rubio-Martínez J. (2023). α4-α5 Helices on Surface of KRAS Can Accommodate Small Compounds That Increase KRAS Signaling While Inducing CRC Cell Death. Int. J. Mol. Sci..

[2] Chen Q., Jin J., Guo W., Tang Z., Luo Y., Ying Y., Lin H., Luo Z. (2022). PEBP4 Directs the Malignant Behavior of Hepatocellular Carcinoma Cells via Regulating mTORC1 and mTORC2. Int. J. Mol. Sci..

[3] Kopra K., Valtonen S., Mahran R., Kapp J.N., Hassan N., Gillette W., Dennis B., Li L., Westover K.D., Plückthun A. (2022). Thermal Shift Assay for Small GTPase Stability Screening: Evaluation and Suitability. Int. J. Mol. Sci..

[4] Vuelta E., Ordoñez J.L., Sanz D.J., Ballesteros S., Hernández-Rivas J.M., Méndez-Sánchez L., Sánchez-Martín M., García-Tuñón I. (2022). CRISPR/Cas9-Directed Gene Trap Constitutes a Selection System for Corrected BCR/ABL Leukemic Cells in CML. Int. J. Mol. Sci..

[5] Alcoceba M., García-Alvarez M., Medina A., Maldonado R., González-Calle V., Chillón M.C., Sarasquete M.E., González M., García-Sanz R., Jiménez C. (2022). MYD88 Mutations: Transforming the Landscape of IgM Monoclonal Gammopathies. Int. J. Mol. Sci..

[6] Sobolev V.V., Khashukoeva A.Z., Evina O.E., Geppe N.A., Chebysheva S.N., Korsunskaya I.M., Tchepourina E., Mezentsev A. (2022). Role of the Transcription Factor FOSL1 in Organ Development and Tumorigenesis. Int. J. Mol. Sci..

[7] Kasprzak A. (2022). Role of the Ghrelin System in Colorectal Cancer. Int. J. Mol. Sci..

[8] Chénais B. (2022). Transposable Elements and Human Diseases: Mechanisms and Implication in the Response to Environmental Pollutants. Int. J. Mol. Sci..

[9] Mombiela R.M., Arildskov A.R., Bruun F.J., Hasselbalch L.H., Holst K.B., Rasmussen S.H., Borrás C. (2022). What Genetics Can Do for Oncological Imaging: A Systematic Review of the Genetic Validation Data Used in Radiomics Studies. Int. J. Mol. Sci..

[10] Marima R., Hull R., Mbeje M., Molefi T., Mathabe K., Elbagory A.M., Demetriou D., Dlamini Z. (2022). Role of Precision Oncology in Type II Endometrial and Prostate Cancers in the African Population: Global Cancer Genomics Disparities. Int. J. Mol. Sci..

